# Evaluation of mobility parameters in individuals with Parkinson's disease with and without freezing of gait

**DOI:** 10.1055/s-0045-1806821

**Published:** 2025-04-27

**Authors:** Cristiane Ramos de Morais, Tamine T. C. Capato, Ariana Moura Cabral, Adriano de Oliveira Andrade

**Affiliations:** 1 Universidade Federal de Uberlândia, Faculdade de Engenharia Elétrica, Núcleo de Inovação e Avaliação Tecnológica em Saúde, Uberlândia MG, Brazil.; 2 Universidade de São Paulo, Faculdade de Medicina, Departamento de Neurologia, São Paulo SP, Brazil.; 3 Radboud University Medical Centre, Donders Institute for Brain, Cognition and Behavior, Department of Neurology, Centre of Expertise for Parkinson & Movement Disorders, Nijmegen GE The Netherlands.

**Keywords:** Parkinson Disease, Gait, Freezing, Rehabilitation, Mobility Limitation

## Abstract

**Background** Individuals with Parkinson's disease (PD) often experience gait disturbances, and one of the most disabling is freezing of gait (FOG), which is characterized by the inability to initiate or continue walking. To identify its impact on the mobility of PD patients, it is necessary to objectively assess mobility and conduct systematic gait evaluations.

**Objective** To evaluate mobility and gait parameters in individuals with PD with and without FOG.

**Methods** The present cross-sectional study included a group of individuals with PD, divided into those with and without FOG, and a healthy control group. The Timed Up and Go (TUG) test was used to evaluate gait and mobility of the participants. Activities of daily living and motor performance in PD patients were assessed through parts II and III of the Movement Disorder Society Unified Parkinson Disease Rating Scale (MDS-UPDRS), while FOG was assessed through the New Freezing of Gait Questionnaire (NFOG-Q).

**Results** A significant difference between the case and control groups was observed in the mean time it took to perform the TUG test, regardless of FOG, during the off-medication state. Unlike the group without FOG, there was a difference in the time it took to perform the tests between medication states in the group with FOG. Additionally, a correlation was found between TUG time and MDS-UPDRS scores in the FOG group.

**Conclusion** Mobility in PD is influenced by medication effect and the presence of FOG. The results highlight the importance of objective and systematic gait evaluations to identify mobility problems, develop effective rehabilitation strategies, and optimize pharmacological treatments, especially for individuals with FOG.

## INTRODUCTION

Gait impairment is a common symptom in individuals with Parkinson's disease (PD), 1 and gait bradykinesia is characterized by a reduction in step length and speed. 2 The mobility of people with PD is a decisive factor for a good quality of life. 2 3 Furthermore, in the later stages of the condition, patients may experience freezing of gait (FOG), which can lead to falls and be extremely disabling. 4 Characterized by a sudden inability to start or continue walking, patients report FOG as a sensation that their feet are “glued to the ground”. 5 Subjects with FOG present a reduction in mobility and physical and daily activities which, consequently, decreases their participation in society. 3

Certain common situations can trigger FOG, such as when starting to walk, changing directions, passing through narrow spaces, or just before reaching a destination. 4 Moreover, behavior and cognitive changes (non-motor symptoms such as anxiety, stress, and inattention) can also trigger FOG. 6 The mechanisms through which FOG is triggered remain unclear. Therefore, more research is needed in this field for more assertive treatments with long-term effects. 7

To clarify the limiting conditions of mobility in people with PD, it is important to systematically assess the issues that directly interfere with gait patterns, such as FOG. Although numerous studies have used gait assessment scales and mobility tests in PD patients, only a few have specifically addressed functional mobility.

The hypothesis of the current study is that the mobility of participants with FOG would be worse than that of those without FOG when assessed by the Timed Up and Go (TUG) test. This difference would be more pronounced in the off-medication (OFF) state when compared to the on-medication (ON) state. In addition, gait bradykinesia, disease duration, and cognition may have a greater influence on gait patterns and a greater impact on mobility in FOG patients compared to non-FOG subjects. The objective of the present study is to evaluate the gait mobility parameters in PD patients with and without FOG.

## METHODS

### Study design

The current cross-sectional, observational study included a group of PD patients, divided into those with FOG (GFOG + ) and those without it (GFOG-), as well as a control group of healthy volunteers.

### Ethical considerations

The experimental protocol of the present study was approved by the institutional Ethics Committee (under CAAE number: 38885720.3.0000.5152). All participants were fully informed about the study and provided written consent by signing the free and informed consent form.

### Population

The current study included 20 people with idiopathic PD, with and without FOG, and 12 age- and gender-matched healthy individuals, resulting in a total of 32 volunteers. The participants were recruited through convenience sampling from the Associação Parkinson do Triângulo (APT) in the city Uberlândia, state of Minas Gerais, Brazil. All assessments were conducted at the institution.

### Inclusion and exclusion criteria

We included individuals with idiopathic PD diagnosed by a neurologist who: were on stable dopaminergic treatment; had no other associated musculoskeletal or neurodegenerative disease; scored > 15 on the Mini-Mental State Examination (MMSE); and were able to walk at least 10 meters in the OFF state. Participants with any symptoms related to coronavirus disease 2019 (COVID-19), such as fever or cough, were excluded.

### Data collection

**Figure 1:**
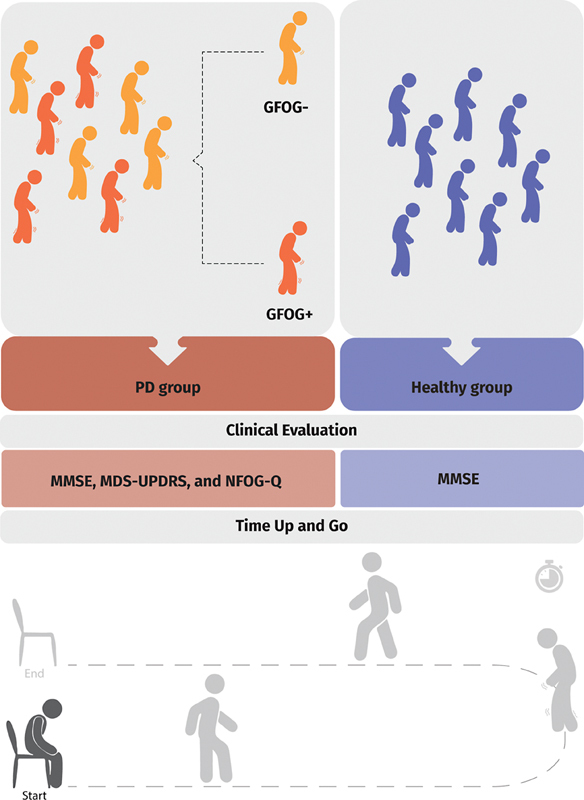
Steps followed during the data collection for the present study.

Figure 1 shows the study design. The clinical evaluation was performed by a physiotherapist with experience in PD. All tests were recorded, and the scores were stored in the Integrated Biomedical Data System (Sistema Integrado de Dados Biomédicos, SIDABI, in Portuguese), developed by the Centre for Innovation and Technology Assessment in Health (Núcleo de Inovação e Avaliação Tecnológica em Saúde, NIATS, in Portuguese), for further analysis.

Initially, a cognitive screening was performed using the MMSE. 8 Then, the participants were assessed in the ON and OFF states through parts II (daily life activities) and III (motor aspects) of the Movement Disorder Society Unified Parkinson Disease Rating Scale 9 (MDS-UPDRS) and the Hoehn and Yahr (H&Y) scale. 10 The New Freezing of Gait Questionnaire (NFOG-Q), 11 Brazilian Portuguese version, 6 was applied to the PD group to quantify FOG severity. Additionally, the TUG test 12 was employed to evaluate mobility and gait speed among PD patients in the ON and OFF states, as well as among the healthy individuals.

The PD subjects were tested during the OFF state in the morning, approximately 12 hours after their last dose of levodopa. The time it took to perform the TUG test was measured across three trials in the OFF state and three trials in the ON state, 30 to 50 minutes after medication use. The mean TUG time was calculated separately for each participant in both medication states.

### Data analysis

In the current study, all statistical analyses were performed using R (R Foundation for Statistical Computing, Vienna, Austria), a programming language and open-source software for data visualization and statistical analysis.

To assess the homogeneity between the different groups in terms of the sex of the participants, the Fisher's exact test was applied. To assess homogeneity in terms of age and cognitive level (MMSE scores), one-way analysis of variance (ANOVA) was performed when this type of approach was considered adequate for comparison, and the Kruskal-Wallis test, when the assumptions of the one-way ANOVA were not met. To verify the assumption of normality of the residuals of the one-way ANOVA, the Shapiro-Wilk test was used, while to evaluate the assumption of homogeneity of variances, the Levene test was used.

To assess homogeneity in terms of the other characteristics of the participants (disease duration, H&Y scores, and MDS-UPDRS scores during the OFF and ON states), the paired *t* -test was applied when normality was verified, and, otherwise, the Wilcoxon test. The Shapiro-Wilk test was used to evaluate the normality of the distributions. In all tests, a significance level of 5% was considered and the *p* -values were adjusted using the Benjamini and Hochberg method.

In addition, the statistical analyses were designed to verify the following:

- Differences between the mean TUG time for the PD patients (GFOG+ and GFOG-) in the OFF state and the volunteers in the healthy group;
- Differences between the mean TUG time for the GFOG+ and GFOG- groups in the ON state;
- Differences in mean TUG test time for the GFOG+ group in the OFF and ON states;
- Differences in the mean TUG test time for the GFOG- group in the OFF and ON states;
- The association between the Mini-Mental score and the TUG test execution time in the OFF and ON medication state;
- The association between NFOG-Q and TUG times in the OFF and ON states;
- The association between MDS-UPDRS scores (parts II and III) and TUG time for the GFOG+ and GFOG- groups in the OFF and ON states; and
- The association between the time until PD diagnosis and the TUG time in the OFF and ON states.

The comparison of the mean TUG time among the three (control, FOG + , and FOG-) groups was performed using one-way ANOVA, considering a significance level of 5%. To verify the validity of the ANOVA, the Shapiro-Wilk and Levene tests were used. When the assumptions of the one-way ANOVA were not met, the Kruskal-Wallis test was employed. For multiple comparisons, the Tukey test was used for the parametric approach, and the pairwise Wilcoxon test, for the non-parametric approach. In all tests, a significance level of 5% was considered, and the *p* -values were adjusted using the Benjamini and Hochberg method.

In addition, the comparison of the mean time to run the TUG test between the FOG+ and FOG- groups was performed using the paired *t* -test when the data followed a normal distribution, or the Wilcoxon test when the data did not. In all tests, a significance level of 5% was considered, and the *p* -values were performed using the Benjamini and Hochberg method.

To examine the associations between the mean TUG times and the clinical scores of FOG+ group in the OFF and ON states, a correlation analysis was also performed, with an estimation of the Pearson's correlation coefficient and considering a significance level of 5%.

For the analysis comparing the PD patients to the healthy individuals, two groups were considered: group A included 20 individuals with PD (10 FOG+ and 10 FOG-), while group B consisted of 12 subjects from the control group.

## RESULTS

### Sample demographics

In total, 32 individuals participated in the present study: 20 PD patients and 12 healthy individuals. Most of the GFOG+ and GFOG- participants in the PD group exhibited moderate disease severity, even during their usual levodopa medication state. Details of the characteristics of the study sample are shown in Table 1 .

**Table 1:** Characteristics of the study sample

Clinical characteristic	GFOG+ (N = 10)	GFOG- (N = 10)	Control group (N = 12)
Age (in years): mean ± SD	61 ± 7	67 ± 6	65 ± 7
Sex: male subjects (%)	40	60	58
Disease duration (in years): mean ± SD	12 ± 6	7 ± 4	
MMSE score (mean ± SD)	25 ± 4	26 ± 2	27 ± 3
Total score on parts II and III of the MDS-UPDRS in OFF (mean ± SD)	74 ± 37	55 ± 18	
Total score on parts II and III of the MDS-UPDRS in ON (mean ± SD)	59 ± 34	42 ± 18	
Score on the Hoehn and Yahr scale (mean ± SD)	2.9 ± 0.88	2.6 ± 0.70	

Abbreviations: GFOG + , group with freezing of gait; GFOG-, group without freezing of gait; MMSE, Mini-Mental State Examination; OFF, off-medication state; ON, on-medication state; SD, standard deviation.

**Figure 2:**
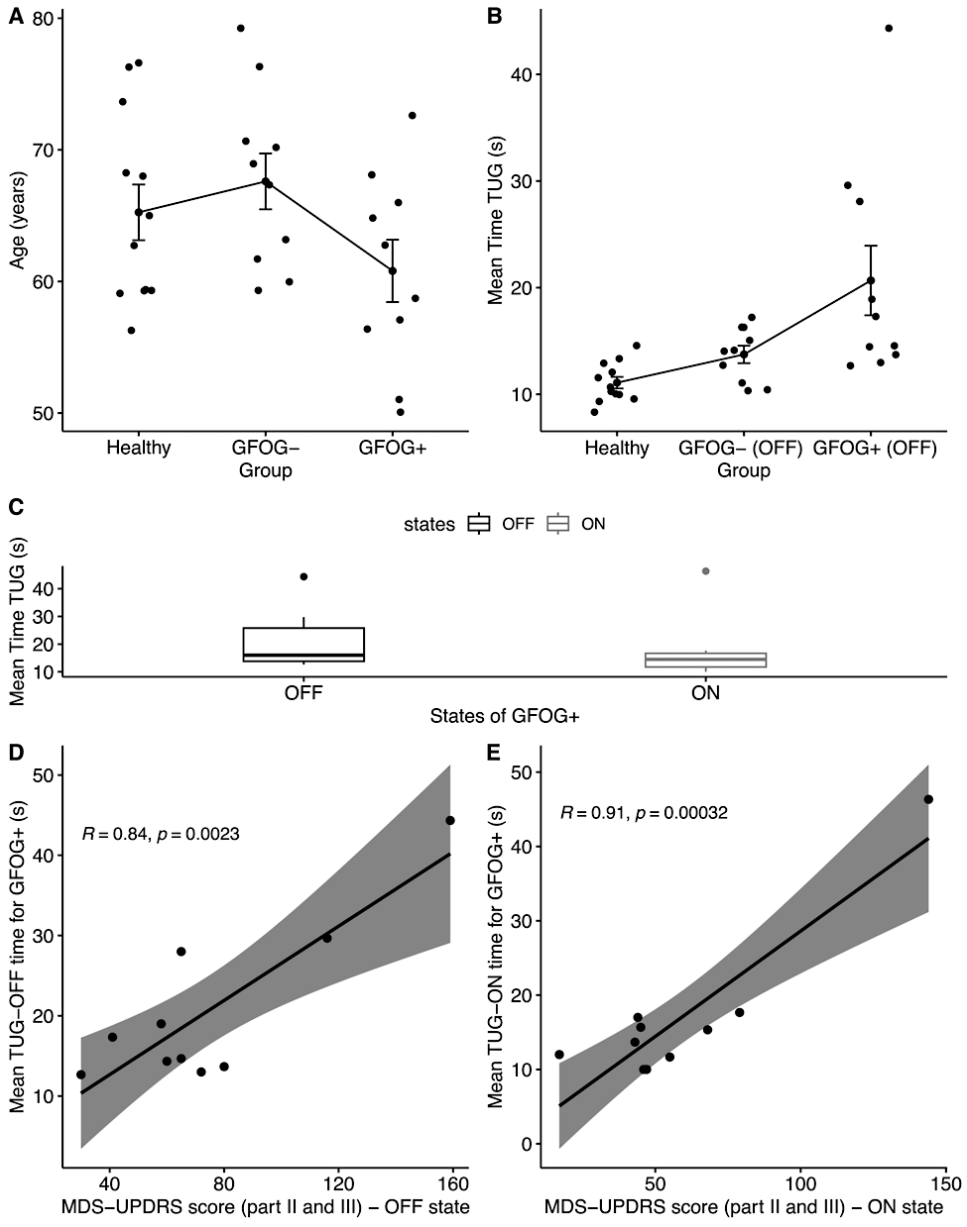
( **A** ) Mean age of the study participants. ( **B** ) Mean time to perform the Timed Up and Go (TUG) test for the case and control groups in the off-medication (OFF) state. ( **C** ) Mean TUG time in the OFF and on-medication (ON) states for the group of PD patients with freezing of gait (GFOG + ). ( **D** ) Mean TUG time in the OFF state for the GFOG + . ( **E** ) Mean TUG time in the ON state for the GFOG + .

There was no statistically significant difference among the three groups in terms of age ( *p* = 0.12), as illustrated in Figure 2A , gender ( *p* = 0.74), or cognitive level ( *p* = 0.43). In addition, the MDS-UPDRS scores, both in the OFF ( *p* = 0.15) and ON ( *p* = 0.10) states, were not statistically different between patients with and without FOG.

Similarly, no statistically significant difference was observed when comparing time until diagnosis ( *p* = 0.93) and disease staging ( *p* = 0.43) in the PD group, regardless of FOG. Additionally, no correlation was found between the age at diagnosis (in years) and the mean TUG time (in seconds) among PD patients in the OFF (r = 0.14; *p* > 0.05) and ON (r = -0.03; *p* > 0.05) states.

### Factors which can impact gait mobility parameters in PD patients with and without FOG

Figure 2B shows the difference in mean TUG test times (in seconds) for the GFOG+ and GFOG- groups in the OFF state and the control group ( *p* < 0.05). No significant difference was found between the mean TUG times in the ON state for the GFOG- and GFOG+ groups ( *p* > 0.05). Figure 2C shows a significant difference in mean TUG times between the OFF and ON states for the GFOG+ group (r = 0.14; *p* < 0.05), with the highest values observed in the OFF state. The difference in the mean TUG time was of 3.7 seconds.

When we compared the results of the TUG test in the OFF and ON states for the GFOG- group, no significant correlation was found (r = 0.02; *p* > 0.05). Additionally, there was no correlation between MMSE scores and TUG times in the PD group in the ON (r = 0.11; *p* > 0.05) and OFF (r = -0.05; *p* > 0.05) states. Similarly, no correlation was observed between NFOG-Q scores and mean TUG times in the OFF (r = 0.46; *p* > 0.05) and ON (r = 0.37; *p* > 0.05) states for the individuals with PD.

For the GFOG+ group there was a correlation regarding the mean TUG times in the OFF (r = 0.84; *p* < 0.05) and ON (r = 0.90; *p* < 0.05) states and the sum of the score on parts II and III of the MDS-UPDRS ( Figure 2D,E ). However, this correlation was not observed for the GFOG- group in the OFF (r = 0.41; *p* > 0.05) and ON (r = 0.29; *p* > 0.05) states.

## DISCUSSION

The findings of the current study supported several of our hypotheses. First, significant differences were observed regarding the gait mobility parameters of the case and control groups. The PD patients presented slower gait speeds in the TUG test, which is in line with the results of previous studies. 13 It is expected that individuals with longer TUG performance also face additional challenges, including festination and FOG, 14 15 balance issues including falls, and significant impairments in their daily activities. 16

Notably, the GFOG+ group took longer to perform the TUG in the OFF state, while levodopa (ON state) significantly improves their performance in the test. Despite observing a significant improvement in the ON state, this difference was not observed in the GFOG- group. These results reinforce the effectiveness of levodopa in enhancing gait, especially in the reduction in FOG episodes and FOG duration (time spent freezing) and in the increase in gait-specific spatio-temporal parameters. 17 Our results also suggest that levodopa helps reduce the frequency and duration of FOG. Previous studies 18 have also shown a significant effect of medication on gait speed. However, it is well known that dopaminergic therapy only partially improves gait impairments in individuals with PD who experience FOG, and management is still a challenge. 19

In the present study, we did not observe any associations regarding the TUG time and the scores on the MMSE and NFOG-Q. On the one hand, the absence of correlation with MMSE scores may be attributed to the inclusion of participants with relatively-preserved cognitive function. 20 In addition, the lack of correlation with the NFOG-Q scores may be due to the inherent limitations of the clinical instrument. 21 The NFOG-Q only assesses the occurrence and duration of FOG episodes, specifically at step initiation and during changes of direction. 18

There was a strong statistically significant correlation regarding the TUG time in the GFOG+ group and the MDS-UPDRS scores (on parts II and III). This indicates that longer TUG times reflect poorer functionality and increased risk of falling. It is well known that, during FOG episodes, individuals with PD may experience a loss of balance and stability, leading to tripping or stumbling, which can result in falls and fall-related injuries. 16 This relationship underscores the importance of evaluating gait and motor impairments in PD patients, especially those with FOG, as these factors significantly impact balance. 7

Interestingly, no association was observed between the mean TUG time and disease duration, suggesting that the severity of gait symptoms is not solely related to PD progression; it is also multifactorial. 3 Undoubtedly, other disabling symptoms can also influence gait, which may be related to the phenotype of the pathology, psychological factors, and the individual's profile and functional level. 22 Other factors, such as muscle strength, coordination, balance, and individual patient profiles, can also influence gait. This finding highlights the complexity and variability of gait impairments in PD, emphasizing the need for personalized assessment and intervention by healthcare professionals to effectively address specific mobility challenges. 3

Analyzing gait and mobility in patients with PD is challenging due to the heterogeneity of signs and symptoms observed. Therefore, it is essential for healthcare professionals, including physiotherapists and occupational therapists, to identify the specific factors impacting mobility for each patient individually. This personalized approach enables the prescription of interventions aimed at improving gait-related issues, such as FOG, to maintain and enhance mobility.

Although the current study recruited a relatively small number of participants, the results found were consistent with those reported in the literature, considering that a worsening of gait-related parameters was found in participants with PD, with or without FOG, when compared to healthy individuals ( Figure 2 ). However, due to the sample size and homogeneity among the groups compared in terms of different demographic and clinical characteristics, the results presented do not enable us to generalize the gait profile of the entire Brazilian population with PD, given that there is a great variability of motor signs among PD patients. In addition, the present study did not fully consider motor fluctuations due to the effect of medication. Thus, future research should reconfirm these findings by conducting large-scale studies, considering the fluctuations due to the effect of the medication, and including new technologies to further improve the sensitivity of the TUG test.

### Future directions

Wearable technologies, such as inertial measurement units, can complement the gait analysis conducted in the current study by providing a quantitative approach to monitoring and analyzing mobility in PD patients.

In conclusion, the TUG test is an excellent choice to assess the functional mobility of PD patients with and without FOG, for it is sensitive to the OFF and ON states. As it correlates with functional issues reflected in the MDS-UPDRS, it can be a good parameter to evaluate rehabilitation therapy interventions in these subgroups, given its ease of application in the clinical practice. Systematic assessments of gait adopting objective measures are extremely important for PD to identify mobility problems and assist the choice of gait rehabilitation strategies that could be associated with the medical treatment of PD.
